# Blockade of Neuronal α7-nAChR by α-Conotoxin ImI Explained by Computational Scanning and Energy Calculations

**DOI:** 10.1371/journal.pcbi.1002011

**Published:** 2011-03-03

**Authors:** Rilei Yu, David J. Craik, Quentin Kaas

**Affiliations:** Division of Chemistry and Structural Biology, Institute for Molecular Bioscience, The University of Queensland, Brisbane, Queensland, Australia; University of Houston, United States of America

## Abstract

α-Conotoxins potently inhibit isoforms of nicotinic acetylcholine receptors (nAChRs), which are essential for neuronal and neuromuscular transmission. They are also used as neurochemical tools to study nAChR physiology and are being evaluated as drug leads to treat various neuronal disorders. A number of experimental studies have been performed to investigate the structure-activity relationships of conotoxin/nAChR complexes. However, the structural determinants of their binding interactions are still ambiguous in the absence of experimental structures of conotoxin-receptor complexes. In this study, the binding modes of α-conotoxin ImI to the α7-nAChR, currently the best-studied system experimentally, were investigated using comparative modeling and molecular dynamics simulations. The structures of more than 30 single point mutants of either the conotoxin or the receptor were modeled and analyzed. The models were used to explain qualitatively the change of affinities measured experimentally, including some nAChR positions located outside the binding site. Mutational energies were calculated using different methods that combine a conformational refinement procedure (minimization with a distance dependent dielectric constant or explicit water, or molecular dynamics using five restraint strategies) and a binding energy function (MM-GB/SA or MM-PB/SA). The protocol using explicit water energy minimization and MM-GB/SA gave the best correlations with experimental binding affinities, with an R^2^ value of 0.74. The van der Waals and non-polar desolvation components were found to be the main driving force for binding of the conotoxin to the nAChR. The electrostatic component was responsible for the selectivity of the various ImI mutants. Overall, this study provides novel insights into the binding mechanism of α-conotoxins to nAChRs and the methodological developments reported here open avenues for computational scanning studies of a rapidly expanding range of wild-type and chemically modified α-conotoxins.

## Author Summary

Conotoxins are peptide toxins extracted from the venom of carnivorous marine cone snails. Members of the α-conotoxin subfamily potently block nicotinic acetylcholine receptors (nAChRs), which are involved in signal transmission between two neurons or between neurons and muscle fibers. nAChRs are important pharmacological targets due to their involvement in the transmission of pain stimuli and also in numerous neurone diseases and disorders. Their potency and specificity have led to the development of α-conotoxins as drug leads, and also to their use in the investigation of the role of nAChRs in various physiological processes. The most studied conotoxin/nAChR system, ImI/α7, was modeled in this study, and several computational methods were tested for their ability to explain the perturbations observed experimentally after introducing single point mutations into either ImI or the α7 receptor. The aim of this study was to establish a theoretical basis to rapidly identify new α-conotoxin mutants that might have improved specificity and affinity for a given receptor subtype. Furthermore, hundreds of thousands of conotoxins are predicted to exist, and computational methods are needed to help streamline the discovery of their molecular targets.

## Introduction

**Figure 1:**
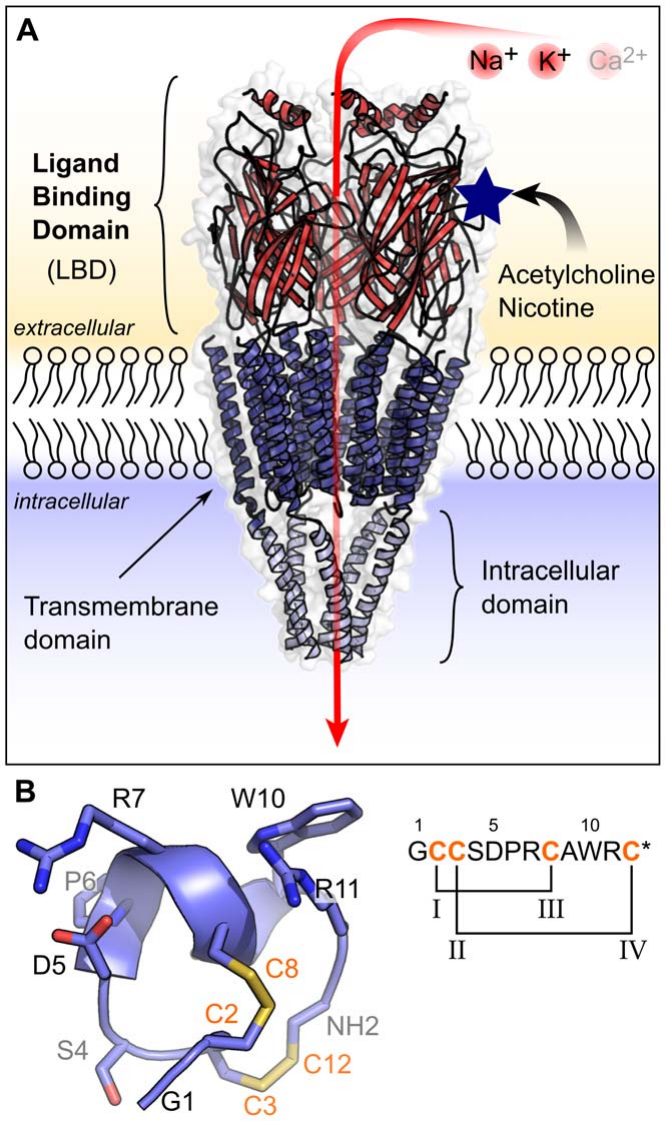
Nicotinic acetylcholine receptor structure and α-conotoxin ImI. (A) Nicotinic acetylcholine receptors (nAChRs) are ligand gated-ion channels. Their structure is composed of a ligand-binding domain (red), a transmembrane domain (blue), and an intracellular domain (white). nAChRs are permeable to Na^+^ and K^+^ and, for some isoforms, Ca^2+^. The opening of the channel is triggered by acetylcholine or nicotine. One of the acetylcholine binding sites is indicated as a blue star. (B) α-conotoxin ImI comprises 12 residues and is C-terminally amidated (indicated by * in the sequence). The structure features a short α-helix and two disulfide bonds that link cysteines I-III and II-IV.

Nicotinic acetylcholine receptors (nAChRs) are a large family of ligand-gated ion channels that mediate rapid synaptic transmission in the central and peripheral nervous system [1], [2]. nAChRs are implicated in disorders such as Alzheimer's diseases, schizophrenia, depression, hyperactivity disorders and tobacco addiction [3]–[6]. All nAChRs are comprised of five homologous subunits, which are divided into a large N-terminal extracellular ligand-binding domain (LBD), a transmembrane domain, and an intracellular domain [7] (Figure 1). The nAChR subtypes include hetero- or homo-pentamers of α1-10, γ, β1-4, δ and/or ε subunits. These subtypes differ in their pharmacological and kinetic properties, as well as their localization [8], [9]. For example, the α7-nAChR is widely expressed in the brain, whereas the α3β2-nAChR is mostly expressed in the cerebellum and spinal cord [10].

Conotoxins are disulfide-rich toxins produced in the venom gland of marine cone snails [11], [12]. Each of the >500 species in the Conus genus produces hundreds of different conotoxins [13]–[15], which together form a large pool of many thousands of bioactive peptides. Conotoxins target a diverse range of membrane receptors and ion channels to rapidly and efficiently immobilize prey [13]. The α-conotoxin family specifically and potently inhibits nAChR subtypes and, consequently, these conotoxins are useful tools in neurophysiological studies. The ability to specifically target nAChRs has also attracted interest for the development of drugs, and several conotoxins or derivatives are currently in clinical trials for the treatment of pain [16], [17]. The majority of known α-conotoxins display a similar topology, as shown in Figure 1. This topology includes four cysteines arranged in a common sequence pattern -CCX*_m_*CX*_n_*C-, where X is any non-cysteine residue, and *n* and *m* are the numbers of inter-cysteine residues. Disulfide bonds connect cysteines I-III and II-IV [18], [19].

ImI is one of the shortest α-conotoxins, with a loop spacing topology of *m*  = 4, *n* = 3 [20] and, initially, was reported to specifically interact with α7- and α9-nAChRs [21]. Later, the α3β2-nAChR was also found to be blocked by ImI [22]. ImI has been extensively studied: its structure has been determined using NMR [23]–[25], and its interaction with the α7-nAChR has been probed by several mutational studies [26]–[31]. In the absence of a crystallographic structure of any nAChR, several early structural models of the binding of ImI to the LBD of α7-nAChR were generated [22], [32], but they are now superseded because better templates, additional experimental data and improved modeling methods are available [33]–[35].

In this study, an improved model of the interaction of α7-nAChR with wild-type ImI has been developed and the structural and energetic impact of more than 30 mutations of ImI and of selected positions of the receptor were investigated. We describe for the first time a model able to explain the majority of mutation studies. Optimal methods to predict relative mutational energies were investigated, and an approach that used energy minimization produced excellent correlations with experimental values, producing R^2^ values of 0.74. Finally, an energy decomposition of the mutational energies was done and showed that different terms of the energy function played distinct roles. Although we focus here on conotoxin ImI, experimental mutational studies have been carried out on a range of other conotoxins, in a first step toward their development as drugs [30], [31], [36]. *In silico* mutational studies such as those described here could dramatically accelerate the development of conotoxin-based drugs and also help identify wild-type toxins with interesting pharmacological activity among the thousands of conotoxins that are predicted to exist.

## Results/Discussion

**Figure 2:**
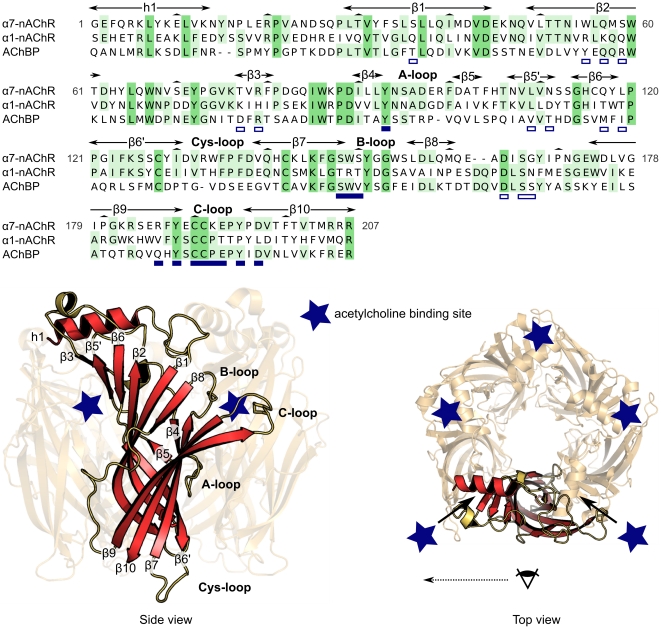
Sequence and structure of α7-nAChR ligand-binding domain. Sequence alignment of *Homo sapiens* α7-nAChR ligand-binding domain (LBD) (UniProtKB/SwissProt P36544), *Mus musculus* α1-nAChR LBD (PDB ID: 1pq1), and *Aplysia californica* AChBP (PDB ID: 1tg9), which is structurally analogous to nAChRs. Below the alignment, the secondary structure elements and acetylcholine binding sites are shown on the lowest energy three-dimensional model of the α7-nAChR nAChR LBD obtained by comparative modeling. Residues in the sequence alignment are numbered according to the α7-nAChR sequence. The conserved positions between the three sequences are on a dark green background, whereas the positions presenting amino acids shared by only two sequences are on a light green background. The secondary structure elements are the α-helix h1 and the β-strands β1-10. The LBD is a pentamer of five subunits. The acetylcholine binding sites, indicated by star symbols, are located at the interface between the subunits. These binding sites mainly comprise the C-loop from one subunit, which is designated as the principal subunit, and the beta strands β1, β2, β3, β5′ and β6 from another subunit, which is designated as the complementary subunit. The secondary structures of one subunit are highlighted in the side view, and the arrangement of the subunits and of the binding sites is shown on the top view. In the alignment, the residues of AChBP in contact with ImI in the crystal structure 2c9t are underlined in blue for positions in the principal subunit and in white for positions in the complementary subunit.

In the first step of this study a model of the complex between ImI and the human α7-nAChR LBD was generated. The crystallographic structures of ImI bound to an *Aplysia californica* acetylcholine binding protein (AChBP), which is distantly related to nAChRs, (PDB ID: 2c9t) [34] and of bungarotoxin bound to the LBD of an isolated subunit α1 (PDB ID: 2qc1) [35] were used to build the initial model, using comparative modeling. Another crystallographic structure of ImI bound to AChBP (PDB ID: 2byp) [33] has been determined but was not used as a template because the coordinates of some amino acid atoms are missing. The secondary structure elements and the location of ImI binding site in our model are displayed in Figure 2 on the sequence and on the lowest energy model of α7-nAChR. This model was then subjected to 10 ns of molecular dynamics simulation. Thirty-four single point mutations of ImI/α7-nAChR that have been experimentally described in previous studies [26]–[30] were then generated in a series of models extracted over the 10 ns simulation. Finally, 14 different strategies were compared to evaluate the mutational energies of single point mutants.

### Conformational variability of α7-nAChR in apo state and bound to ImI

**Figure 3:**
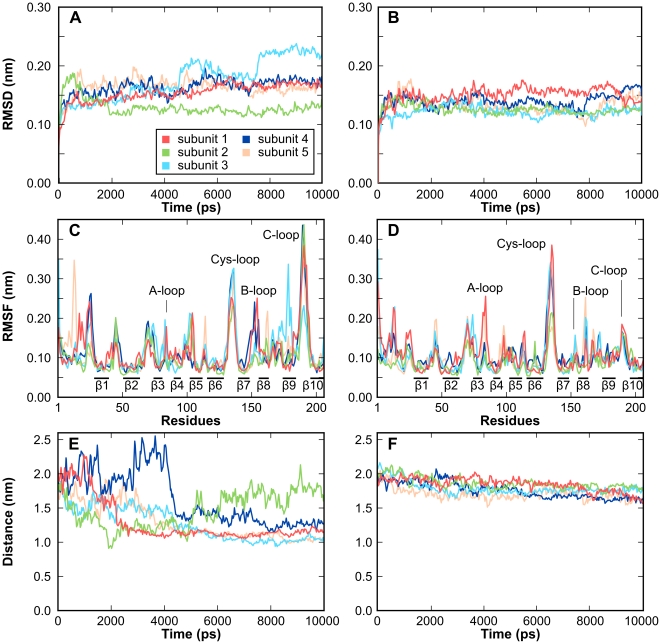
Analysis of the stability of α7-nAChR over 10 ns molecular dynamic simulations in the apo (A,C,E) and ImI-bound (B,D,F) states. β strand α carbon root-mean-square deviations (RMSD) of each of the subunits over the molecular dynamics simulations to the starting frame for the apo (A) and ImI-bond models (B). α carbon root-mean-square fluctuation (RMSF) of each subunit of the apo (C) and ImI-bond (D) models. Fluctuation of the distance between the sulfur atom of α7-C190 side chain and the α carbon of α7-Y32 in the apo (E) and ImI-bond (F) models. This distance characterizes the closure of the C-loop. The RMSD is calculated using Cα atoms in β strands. The RMSD and distances were averaged using a 16 ps window.

Two series of 10 ns molecular dynamics simulations of the α7-nAChR, either in the apo state or bound to ImI, are summarized in Figure 3. The α carbon root-mean-square deviation (RMSD) to the initial conformation became stable after 2000 ps for both simulations, indicating that they had reached equilibrium (Figure 3A,B). Indeed, the largest fluctuation, which is displayed by the third subunit, is <1 Å over the last 8000 ps of the simulation. The α-carbon root-mean-square fluctuations (RMSF) indicate that the β-strand regions are conformationally stable, but that the C-loop and Cys-loop regions are flexible (Figure 3C,D). The dynamic property of the C-loop is particularly interesting, as the change of conformation of this loop is thought to be vital for the physiological role of nAChRs [33], [37]–[40]. It has been shown that the interaction of agonists with nAChRs causes the C-loop to adopt a closed conformation and this change of conformation has been hypothesized to trigger the opening of the channel [41]. According to this hypothesis, competitive antagonists stabilize the C-loop in an open conformation, potentially preventing the channel from opening. Interestingly, in our study, the C-loop in the apo model fluctuates significantly (Figure 3E), whereas the C-loop of the α7-nAChR in complex with ImI is stabilized in an open conformation (Figure 3F). It can therefore be concluded that ImI stabilizes the C-loop in an open conformation, which, according to previous studies, should inhibit channel activity.

**Figure 4:**
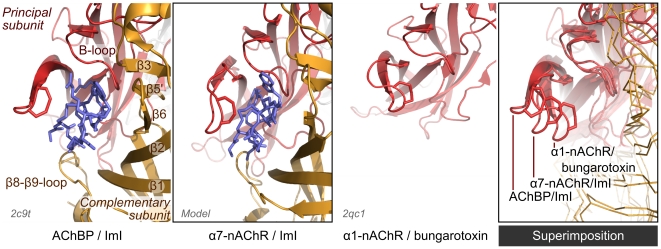
Comparison of the binding site of AChBP/ImI complex (PDB ID: 2c9t), α7-nAChR/ImI complex (model, this work) and α1-nAChR/bungarotoxin complex (PDB ID: 2qc1). In the α1-nAChR/bungarotoxin structure, only one subunit was crystallized, and the bungarotoxin is not shown. The model displaying α7-nAChR was obtained by a combination of comparative modeling and molecular dynamics, and the displayed conformation corresponds to energetically minimized frames after 10 ns of simulations. The C-loop, the principal subunit, and the complement subunit are indicated. In the three first panels and from left to right, the conformation of the C-loop increasingly reduces the volume of the binding site. The fourth panel, on the right, shows a superimposition of the AChBP and nAChR subunits, highlighting the different C-loop conformations between the model and the two experimental templates.

Molecular dynamics simulation significantly refined the conformation of the α7-nAChR/ImI model. Indeed, after 10 ns molecular dynamics, the conformation of the C-loop of the α7-nAChR/ImI model is stable and different from that of the two templates. As shown in Figure 4, the C-loop of the α7-nAChR/ImI is more closed than the C-loop of AChBP in complex with ImI but more opened than that of α1-nAChR subunit in complex with α-bungarotoxin, which is a classical antagonist of nAChR. The positions of the β-sheets are conserved between the template AChBP crystal structure and the α7-nAChR/ImI model. The h1 α-helices occupy slightly different positions, with the α7-nAChR α-helices being closer from the center of the pore than the AChBP ones (not shown).

### Comparison to previous modeling and pairwise interaction studies

**Figure 5:**
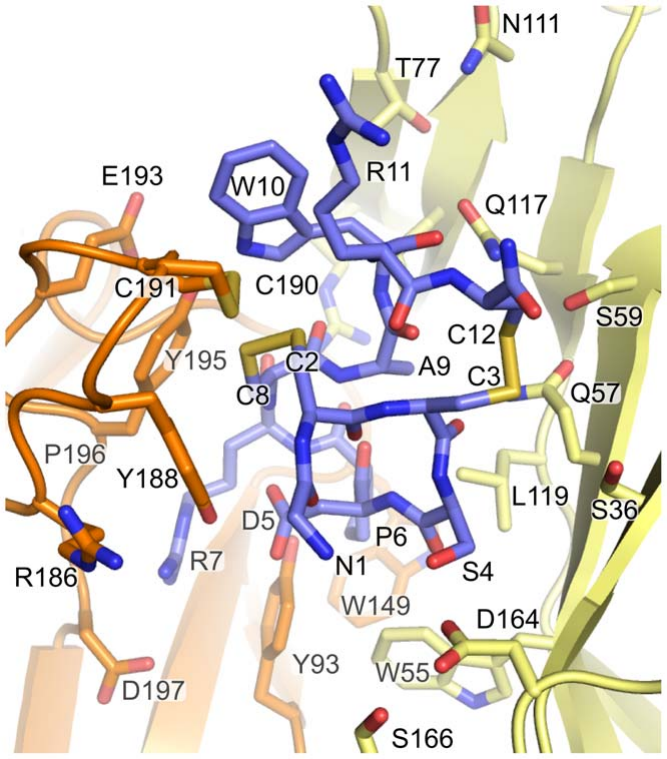
Analysis of the binding mode of ImI to the α7-nAChR. The structure of the binding pocket occupied by ImI after molecular dynamics simulation is displayed and positions discussed in the text are highlighted. The α7 principal subunit is in orange, the α7 complementary subunit is in pale yellow, and ImI is in violet. Nitrogens are in blue, oxygens are in red and sulfurs are in yellow.

Our model of α7/ImI significantly differs from those [22], [32] that were developed before the publication of the crystallographic structures of AChBP/ImI [33], [34]. In the previous studies, models were built by homology with crystallographic structures of AChBP with the C-loop in a closed conformation, but several recent studies suggest that this C-loop conformation is incompatible with the nAChR inactive state [38], [41]. Moreover, the previous studies tentatively tried to justify the binding mode of ImI using weak mutational energy couplings revealed by mutant cycle analyses, which were interpreted as pairwise interactions [28]. It proved to be impossible to reproduce all the pairwise interactions identified by this method [28]. Recently, Gleitsman *et al.* [42] measured similar weak mutational energy couplings occurring between residues located 60 Å from each other, one being in the C-loop of an nAChR and the other in the middle of the trans-membrane domain. That study demonstrated that weak couplings are not evidence of direct interaction. On the contrary, a strong coupling was observed between α7-Y195 and ImI-R7 [28], and in our model, the side chains of these two residues are tightly packed together, as is apparent in Figure 5.

Recently Armishaw *et al.* [30] docked ImI into a structural model of the α7-nAChR derived by comparative modeling, using one of the AChBP/ImI crystallographic structures. Their strategy involved the mutation of α7-Y93 to Ala before performing the docking procedure, and finally the “back” mutation of position 93 into Tyr. Presumably, the docking strategy did not succeed to place the conotoxin without this mutation step. Indeed, docking molecules onto a structure derived by comparative modeling is a challenging task because the low accuracy of the receptor conformation either causes steric hindrance or does not allow side chains to be tightly packed around the docked ligand [43]. The model presented by Armishaw *et al.* [31] is very similar to the final conformation of our molecular dynamics, despite the use of different strategies. Their model was not compared to previous experimental mutation studies, but we here provide qualitative and qualitative explanations to those mutation studies.

### Structural explanation of mutational studies

The binding of ImI to the α7-nAChR has been investigated experimentally and the impact of mutations of α7 and/or ImI on the affinity (K_d_) or inhibition activity (IC_50_) are known [26]–[29]. Here we investigate structural explanations for the influence of single point mutations on α7/ImI affinity through an analysis of models of the mutated complex. Mutations involving unnatural residues have not been considered here because their parameters are less refined than those for standard amino acids. The aim of our study is to compare different methods to predict the impact of single point mutations on binding affinities between conotoxin ImI and α7-nAChR; the use of unnatural residues would complicate the interpretation of those comparisons as the deviation between computed and experimental mutational energies could arise from inaccuracy in the parameters as well as from the methodological differences. The α7/ImI model will be referred to as the “wild-type model”, whereas the models of the complexes presenting mutations are referred to as “mutated models”. Three positions of ImI, i.e., D5, P6, and R7, have been found experimentally to be important for the interaction [26]. Four receptor positions, α7-N111, α7-Q117, α7-P120 and α7-153, have some influence on the affinity of the complex but are not directly in contact with ImI in our model [28].

#### ImI-D5

**Figure 6:**
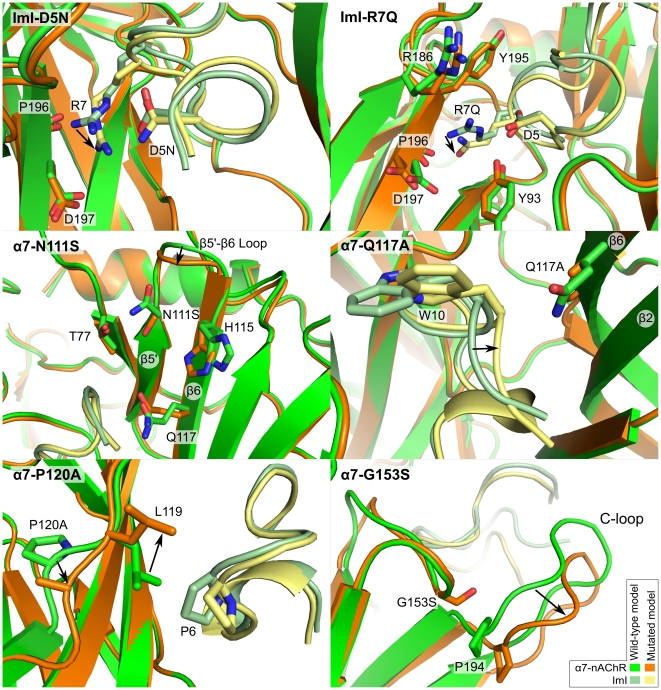
Superimposition of wild-type and mutated models. The models of the mutants shown were refined using molecular dynamics and the conformations shown in this figure are the last frames of the molecular dynamics trajectories. The arrows highlight local conformational changes.

The mutation of ImI-D5 to Asn experimentally decreases the affinity of the complex [27]–[29]. Residues ImI-D5, ImI-R7, α7-D197 and α7-P196 are proximate in the wild-type model, as shown in Figure 6. ImI-D5 is probably involved in charge and hydrogen bond interactions with ImI-R7, which is in turn possibly involved in both a charge and hydrogen bond interactions with the side chain of α7-D197 and the backbone of α7-P196. In the mutated model ImI-D5N, displayed in Figure 6, the side chain of ImI-R7 does not contact α7-D197 and α7-P196 as it does in the wild-type model. Presumably, ImI-D5 plays a significant role to stabilize the conformation of ImI-R7, which allows ImI-R7 to interact with both α7-D197 and α7-P196. Thus, the disruption of the interaction between ImI-D5 and ImI-R7, which is not at the interface, indirectly causes a decrease in affinity by weakening interface interactions between ImI-R7, α7-P196 and α7-D197.

#### ImI-P6

According to our model, ImI-P6 is tightly packed in a hydrophobic pocket formed by α7-W55, α7-Y93, α7-L119 and α7-W149 (Figure 5). Mutations of α7-Y93, α7-W149 and to a lesser extent α7-L119 to a partly hydrophobic Thr residue decreased ImI affinity, and accordingly, the mutated models displayed reduced packing around ImI-P6 (not shown). Consistent with this analysis, an increase in affinity was achieved by introducing a Pro derivative with increased hydrophobicity [30]. Mutations of ImI-P6 to Gly, Ala or Val reduced the affinity of ImI for α7 [26], [28], which is also consistent with the models. However, caution should be exercised in the interpretation of those mutations, as the mutation of P6 induces dramatic conformational changes of ImI backbone [44]. The method we used to model the mutated models cannot take into account such dramatic conformational changes, and therefore the study of ImI-P6 mutants was not carried out.

#### ImI-R7

In the wild-type model, the aliphatic part of the ImI-R7 side chain is in contact with α7-Y93, α7-Y195 and ImI-P196, whereas the positively charged guanidinium moiety is proximate to the negatively charged headgroups of α7-D197 and ImI-D5 (Figure 6). Mutation of ImI-R7 to Gln breaks the charge interactions with α7-D197 and ImI-D5, which is consistent with the decrease in affinity observed experimentally [28]. Moreover, as discussed previously, the mutation of ImI-D5 to Asn also influences the conformation of R7. The mutation of α7-D197 to Asn decreases the affinity for ImI [28], corresponding to a loss of charge interaction (not shown).

The involvement of α7-Y195 in van der Waals interactions with R7 is supported by the observation that α7-Y195T decreases the binding affinity, whereas α7-Y195F does not [28]. An interaction between ImI-R7 and α7-Y195 has also been deduced by double mutant cycle analysis [28].

Mutations of α7-R186 to Ala, Glu, Gln and Val increased the affinity [28]. In the wild-type model, a charge repulsion interaction occurs between α7-R186 and ImI-R7 (Figure 6), and this unfavorable interaction is removed by mutating position 7 into a non-charged or negatively charged residue.

#### α7-N111

α7-N111 is the last position of the β5′-strand and is not in direct contact with ImI. The model of the mutant α7-N111S (Figure 6) features a longer β6 strand and a change in conformation of the β5′-β6 loop compared to the wild-type. Several side chains located in the binding site, including R79, Q117 and H115, have a slightly different orientation in the mutated models. Although our models show that position 111 has an influence on the binding of ImI, it is difficult to provide simple qualitative explanations of the increase in affinity measured experimentally [28].

#### α7-Q117

Mutation of α7-Q117 to Ala or Ser increases affinity for ImI [28]. The α7-Q117A model shows that the conotoxin is closer to the backbone of the β5′ and β6-strands compared to the wild-type (Figure 6) and the buried surface area is increased by 80 Å^2^. This mutation decreases the size of side chain at position 117 and therefore allows ImI-W10 to have better packing at the interface. The increased affinity resulting from the mutation α7-Q117S, which also decreases the size of 117 position side chain, is explained similarly.

#### α7-P120

Mutation of α7-P120 to Ala decreases the affinity [28]. As shown in Figure 6, mutation of α7-P120 to Ala caused a local conformational change of the backbone, which resulted in the rearrangement of the neighboring side chain at position 119. In the wild-type model, the side chain of α7-Ile-119 closely stacks with ImI-P6, but in the mutated model the side chain of α7-Ile-119 has fewer contacts with ImI. Thus, α7-P120 indirectly contributes to the affinity by influencing the conformation of a side chain at the interface.

#### α7-G153

Mutation of position α7-153 from a Gly to a Ser, a bulkier residue, results in a drastic decrease in affinity [28]. In the mutated model the C-loop adopts a more open conformation than in the wild-type model. It is likely that a steric exclusion between (7-S153 and (7-P194 forces the C-loop to change its position relative to the binding site, decreasing the number of interactions at the interface, and therefore accounting for the drop in affinity measured experimentally.

### Comparison of methods to compute mutational energies

Mutational energies of single point mutants were computed using two energy functions: molecular mechanics generalized Born surface area (MM-GB/SA) and molecular mechanics Poisson-Boltzmann surface area (MM-PB/SA) energy functions. The mutated models were first refined using either the minimization based approach (MBA) or the molecular dynamics simulation based approach (MDBA). For the MBA, mutations were introduced in 15 frames extracted from the wild-type 10 ns molecular dynamics simulation and the mutated models were minimized using either explicit water (EWM) or a distance-dependent dielectric constant (DDDCM). For the MDBA, mutations were created on the model of the last frame of the wild-type 10 ns molecular dynamics simulation, and the mutated models were subjected to 500 ps molecular dynamics. Because the complex showed only small conformational variations during the last 8 ns of the simulation, the last frame can be chosen as representative of the wild-type structure. The energies were averaged on the minimized mutated models for MBA, and on 50 frames extracted from the last 100 ps of the 500 ps molecular dynamics for MDBA.

#### Energy predictions using MBA

**Figure 7:**
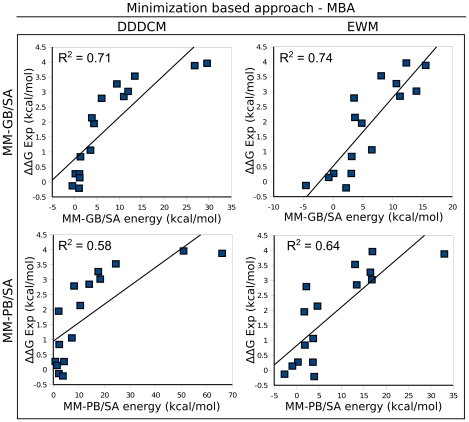
Correlation between experimentally derived and calculated mutational energies of the ImI mutants in the minimization based approach MBA. Mutational energies were computed using either molecular mechanics generalized Born (GB) surface area (MM-GB/SA) or molecular mechanics Poisson-Boltzmann (PB) surface area (MM-PB/SA) energy functions at 298 K. The mutated models were refined using MBA with either distance dependent dielectric constant minimization (DDDCM) or explicit water minimization (EWM). Experimental mutational energies (▵▵G Exp) were derived using the corresponding Kd values of ImI wild-type/(7-nAChR and ImI mutants/(7-nAChR [26]–[28].

The energies of 16 ImI single point mutants were computed (Table 1) and are compared with experimental values in Figure 7. Using the simple DDDCM, MM-GB/SA gave better predictions of mutational energies than MM-PB/SA, as shown by the correlation coefficient R2 of 0.71 and 0.58 between experimentally derived energies and energies computed with generalized Born and Poisson-Boltzmann, respectively. The more computationally intensive EWM led to a subtle improvement for both energy functions, and MM-GB/SA still performed better than MM/PB-SA (R^2^ of 0.74 and 0.64, respectively).

**Table 1:** Calculated and experimental mutational energies (kcal/mol) of ImI mutants.

	MBA^a^				MDBA^b^										
	DDDCM		EWM		(i)		(ii)		(iii)		(iv)		(v)		
Mutant	GB^d^	PB^e^	GB^d^	PB^e^	GB^d^	PB^e^	GB^d^	PB^e^	GB^d^	PB^e^	GB^d^	PB^e^	GB^d^	PB^e^	mutant EXP^c^
S4A	0.2±0.4	0.8±0.5	0.1±0.3	0.3±0.3	3.2±1.2	9.9±1.4	2.5±1.1	10.4±1.1	0.8±0.9	4.9±1.0	−0.3±0.4	−4.4±0.3	4.3±0.3	7.6±0.3	0.3±0.2
D5A	3.8±0.9	10.5±1.5	3.7±0.6	4.7±0.7	3.8±1.0	22.5±1.2	2.0±0.8	10.1±1.4	4.7±1.0	4.5±1.0	4.3±0.4	8.3±0.4	2.2±0.3	4.7±0.2	2.1±0.1
D5K	29.6±3.7	50.9±5.3	12.3±1.6	17.0±1.8	8.3±1.4	20.4±1.6	−0.4±1.2	8.1±1.2	−1.9±1.0	2.2±1.6	37.4±0.6	22.9±0.3	20.6±0.2	12.6±0.3	4.0±0.2
D5N	6.0±0.9	8.1±1.4	3.5±0.8	2.2±0.9	10.9±1.4	23.8±1.4	7.6±1.2	10.6±1.2	6.6±1.2	1.6±1.4	11.8±0.4	10.7±0.4	5.7±0.3	4.0±0.3	2.8±0.2
R7A	11.9±1.3	18.4±1.3	14.0±0.8	16.8±0.9	8.5±1.7	13.2±1.5	10.1±1.3	19.2±1.2	14.5±1.0	15.6±1.0	42.5±0.3	16.4±0.4	15.9±0.3	18.4±0.2	3.0±0.3
R7E	26.8±1.6	66.0±3.6	15.5±1.0	33.0±2.6	0.2±1.3	21.1±1.8	14.4±1.3	43.0±1.3	10.0±1.0	20.3±1.1	36.3±0.4	79.2±0.3	18.1±0.3	36.4±0.4	3.9±0.2
R7K	11.0±1.0	14.0±1.3	11.2±0.8	13.4±1.4	13.3±1.3	16.9±1.3	9.9±1.2	15.7±1.1	9.8±1.0	8.4±1.2	23.1±0.3	26.8±0.4	7.9±0.4	5.5±0.4	2.9±0.2
R7L	9.4±0.8	17.6±1.6	10.6±1.2	16.5±1.2	9.0±1.4	17.9±1.6	5.2±1.0	15.8±1.4	3.0±1.2	6.0±1.3	13.8±0.3	36.2±0.3	9.7±0.3	12.8±0.4	3.3±0.6
R7Q	13.5±1.2	24.5±1.8	8.1±1.2	13.1±1.3	2.0±1.5	15.1±1.3	1.2±1.1	9.7±1.4	6.0±1.0	3.1±1.1	3.3±0.4	5.7±0.4	9.1±0.3	12.1±0.3	3.5±0.2
A9S	−0.5±0.3	2.2±0.7	−4.5±0.8	−2.7±0.9	3.6±1.3	6.0±1.1	−3.2±1.0	−0.7±1.0	−1.6±1.1	−5.1±1.0	−6.8±0.3	−7.0±0.3	−2.3±0.3	−2.8±0.2	−0.1±0.2
W10A	3.5±0.7	7.3±0.8	6.5±0.6	3.7±0.7	7.1±1.4	13.9±1.5	2.8±1.2	8.0±1.2	3.2±1.0	−1.1±1.0	3.0±0.3	−6.9±0.4	6.3±0.3	3.2±0.4	1.1±0.1
W10F	1.3±0.9	2.3±0.4	3.1±0.5	1.9±0.6	−0.5±1.3	7.3±1.4	−2.6±1.2	2.6±1.2	−3.0±1.3	−5.5±1.3	1.7±0.3	−9.3±0.5	5.3±0.4	1.2±0.3	0.8±0.5
W10T	4.3±0.7	2.1±0.6	4.8±0.5	1.7±0.3	4.7±1.3	11.0±1.2	5.8±1.1	4.9±1.1	1.9±1.2	−3.9±1.0	13.9±0.4	4.3±0.4	6.8±0.3	1.6±0.4	2.0±0.2
W10Y	1.1±0.4	1.5±0.5	−0.7±0.7	−0.9±0.9	−0.7±1.2	12.7±1.3	−2.8±1.1	2.2±1.0	−1.1±1.0	0.7±0.8	−14.8±0.3	−23.0±0.4	−0.3±0.2	−3.2±0.2	0.1±0.3
R11A	1.0±0.6	3.8±0.7	2.2±0.8	3.9±0.7	4.0±1.5	6.0±1.2	2.9±1.3	10.2±1.1	−2.8±1.0	−4.5±0.9	−16.2±0.4	−2.8±0.4	1.1±0.3	2.6±0.3	−0.2±0.2
R11Q	1.1±0.6	4.2±0.9	3.1±0.9	3.7±0.7	4.8±1.1	21.3±1.2	4.4±1.0	7.9±0.9	−4.3±1.1	−4.9±0.8	−5.5±0.5	−8.6±0.4	2.7±0.3	2.0±0.4	0.3±0.2
**R^2^**	0.71	0.58	0.74	0.64	0.16	0.40	0.30	0.34	0.44	0.45	0.70	0.59	0.71	0.51	

a Minimization based approach (MBA), which uses either a distance-dependent dielectric constant minimization (DDDCM) or an explicit water minimization (EWM). The standard deviations of the mutational energies were computed using 15 frames.

b Molecular dynamics simulation based approach (MDBA). Five different strategies, noted (i) to (v), were used to refine the conformation of the structural model. (i) all receptor atoms >6 Å from the conotoxin were restrained to their position; (ii) all receptor atoms >4.5 Å from the conotoxin were restrained to their position; (iii) all the atoms located >6 Å from the mutated residue were restrained to their position; (iv) all the atoms from the receptor were restrained to their position, and all the atoms from the conotoxin mutants were free to move; (v) all residues were restrained to their position. The standard deviations of the mutational energies were computed using 50 frames.

c Experimental mutational energies (EXP). The values were derived from experimental K_d_ values [26]–[28] using the equation ΔΔG_binding_  =  EXP  =  −RT ln[K_d_ (mutant)/K_d_ (wild-type)] at 298 K.

d Mutational energy computed using molecular mechanics generalized Born (GB) surface area (MM-GB/SA) at 298 K.

e Mutational energy computed using molecular mechanics Poisson-Boltzmann (PB) surface area (MM-PB/SA) at 298 K.

It is *a priori* surprising that the generalized Born method gave better predictions because generalized Born parameters were optimized to reproduce the more computationally demanding results from finite-difference Poisson-Boltzmann [45]. A possible explanation for this phenomenon is that the approximations introduced in the generalized Born method are able to partly ameliorate the inaccuracy of theoretical models, whereas the Poisson-Boltzmann method cannot. Indeed, it has been shown that MM-GB/SA provides better ranking of models generated by docking than MM-PB/SA [46]–[48]. Few previous studies of protein/peptide complexes used simultaneously MM-PB/SA and MM-GB/SA [49] and to our knowledge none made an extensive comparison of the ability of the two energy functions to rank mutational energies.

Regarding the refinement method, the small superiority of EWM over DDDCM mainly arises from the prediction of two mutational energies, R7E and D5K, which both result in a reversal of charges. Therefore, models minimized using explicit water representation seem to be slightly more accurate that the ones obtained from implicit solvation methods, like DDDCM. Nevertheless, it should be noted that energy computations using DDDCM were, on average, four times faster than EWM.

#### Energy predictions using MDBA

**Figure 8:**
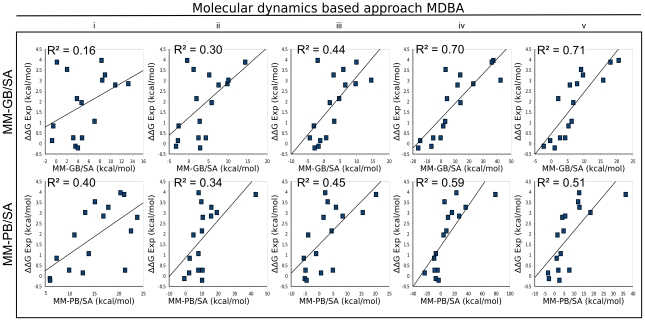
Correlation between experimentally derived and calculated mutational energies of ImI mutants in the molecular dynamics based approach MDBA. Mutational energies were computed by using either molecular mechanics generalized Born (GB) surface area (MM-GB/SA) or molecular mechanics Poisson-Boltzmann (PB) surface area (MM-PB/SA) approaches at 298 K. In the MDBA five alternative position restraint strategies were employed: (i) all receptor atoms >6 Å from the conotoxin were restrained to their position; (ii) all receptor atoms >4.5 Å from the conotoxin were restrained to their position; (iii) all the atoms located >6 Å from the mutated residue were restrained to their position; (iv) all the atoms from the receptor were restrained to their position, and all the atoms from the conotoxin mutants were free to move; and (v) all residues were restrained to their position. Experimental mutational energies (ΔΔG Exp) were derived using the corresponding K_d_ values of ImI wild-type/α7-nAChR and ImI mutants/α7-nAChR [26]–[28].

For the MDBA, short molecular dynamics simulations were carried out to refine the models of the mutants, and the mutational energies were evaluated using MM-GB/SA or MM-PB/SA. The resulting energies are shown in Table 1 and are compared to experimentally derived energies in Figure 8.

To achieve the simulations within a practical computational time, only short simulations could be carried out for each mutant. Because of this short simulation time, the molecular dynamics trajectories only partially sample the accessible conformational space. As a result, the conformations of the positions that are not influenced by the mutations are sampled differently in the simulation of the wild-type and mutant complexes, and those differences create artificial noise in the computation of the mutational energies. To overcome this problem, some atoms were restrained to their initial location by a quadratic force, and five strategies were employed to select the atoms that should be restrained. In strategy (i), all of the receptor atoms located further than 6 Å from the conotoxin were restrained to their position. This led to a poor correlation with experimental values, with R^2^ = 0.16 for MM-GB/SA and R^2^ = 0.40 for MM-PB/SA (Figure 8). To reduce the possible detrimental influence of positions located out of the binding site, additional restraints were added to the system in strategy (ii) by lowering the distance cut-off to 4.5 Å. This change improved the prediction made with MM-GB/SA (R^2^ = 0.30) but worsened the one computed using MM-PB/SA (R^2^ = 0.34). The change in cut-off allowed the decrease in the number of atoms without restraints from about 1000 to 800. In strategy (iii), the mutated residue was taken as the center of the distance cut-off. With a 6 Å distance cut-off, only about 400 atoms were without restraints. The correlation with experimental values improved for both MM-GB/SA and MM-PB/SA compared to strategy (ii), with R^2^ reaching 0.44 and 0.45, respectively. In strategy (iv), all the atoms from the receptor were restrained to their position, but all the atoms from the conotoxin mutants (about 170 atoms) were free to move. Surprisingly, the addition of restraints on the whole receptor significantly improved the correlation of both MM-GB/SA and MM-PB/SA, as the R^2^ reached 0.70 and 0.59, respectively. In the last strategy (v), all residues were restrained, which allowed only subtle changes to the atom positions. The agreement with experimental binding energies was similar to strategy (iv) as R^2^ = 0.71 for generalized Born and R^2^ = 0.51 for Poisson-Boltzmann. By only allowing the atoms to have local moves, the conformational sampling of strategies (iv) and (v) have similar effect to energy minimization, and the correlation with experimental energies are closer to the MBA than strategies (i)-(iii). Nevertheless, despite being considerably more time consuming, the molecular dynamics refinement approach used in the MDBA did not produce a better prediction than the minimization approach used in the MBA. We therefore conclude that the MBA is the best method to use.

#### Mutational energy of receptor mutants

To further validate the accuracy of the MBA, 48 additional mutational energies using DDDCM and EWM were computed by mutating positions of the receptor (Table 2). The correlation coefficient between the experimental and predicted mutational energies does not change significantly by including the predictions made for the receptor mutants (Figures S2). The stability of the correlation coefficient upon addition of new data demonstrate that our models and methods could be used to predict the relative binding affinities of other single point mutations of the ImI/α7-nAChR system.

**Table 2:** Calculated and experimental mutational energies (kcal/mol) of the receptor mutants.

	DDDCM^a^		EWM^b^		
α7 mutant	GB^d^	PB^e^	GB^d^	PB^e^	EXP^c^
L92A	0.7±0.2	0.6±0.3	1±0.5	1.2±0.5	0.4±0.2
Y93T	6.2±1.0	3.7±0.5	6.7±0.5	6.8±0.8	2.2±0.2
S148A	−0.2±0.2	−1.6±0.4	−0.8±0.6	−1.3±0.5	0.5±0.2
W149T	2.0±0.4	2.5±0.7	2.8±0.6	3.2±0.7	2.0±0.2
S150A	0.1±0.1	−0.5±0.4	−0.4±0.5	0.0±0.4	0.0±0.2
R186E	−0.3±0.4	0.1±0.8	−0.2±0.5	−1.0±0.7	−0.8±0.2
R186Q	−0.8±0.3	−1.1±0.7	0.0±0.7	0.3±1.0	−0.8±0.2
R186V	−0.5±0.3	−1.6±0.6	−0.7±0.5	−0.5±0.7	−1.2±0.2
Y188F	−0.5±0.5	−0.6±0.8	−0.7±0.5	−0.5±0.7	0.7±0.2
Y195D	4.8±0.28	10.3±1.0	5.8±0.6	7.1±0.6	2.2±0.2
Y195F	2.2±0.2	2.4±0.4	0.6±0.6	0.0±0.8	0.0±0.2
D197N	6.4±1.0	8.5±1.8	7.1±1.0	8.9±1.5	1.8±0.2
**R^2^**	0.71	0.58	0.64	0.74	

a Distance dependent dielectric constant minimization (DDDCM). The standard deviations of the mutational energies were computed using 15 frames.

b Explicit water minimization (EWM). The standard deviations of the mutational energies were computed using 15 frames.

c Experimental mutational energies (EXP) that were derived from experimental K_d_ values [26]–[28] using the equation ΔΔG_binding_  =  EXP  =  −RT ln[K_d_ (mutant)/K_d_ (wild-type)] at 298 K.

d Mutational energy computed using molecular mechanics generalized Born (GB) surface area (MM-GB/SA) at 298 K.

e Mutational energy computed using molecular mechanics Poisson-Boltzmann (PB) surface area (MM-PB/SA) at 298 K.

### Binding energy and mutational energy decompositions

To better understand the energetic components stabilizing the ImI/α7-nAChR complex, the free energies of the system and the mutational free energies of the mutated complexes, computed using EWM, were decomposed into entropic, electrostatic, van der Waals, and hydrophobic contributions. The solute entropic contribution to the binding energy has been neglected in our previous calculations, but it was estimated using normal-mode analysis in this section. As shown in Table 3, the van der Waals interactions and the hydrophobic effects stabilize the complex, whereas the electrostatic contribution is destabilizing. The observation that the van der Waals interactions and hydrophobic effect are predominant over the electrostatic interactions correlates with a statistical analysis of interface features carried out over the 10 ns of the molecular dynamics simulation. During the simulation, the average buried surface area of the wild-type complex was of 1150 Å^2^, which is twice as large as the average 500 Å^2^ of peptide/protein interfaces [50], and can be associated with the important van der Waals and hydrophobic effect energies. ImI and α7-nAChR form, on average over the simulation, three hydrogen bonds and one salt-bridge, and this small number of electrostatic interactions is consistent with average values for α-helical peptides [50].

**Table 3:** Decomposition of the binding free energy (kcal/mol) of ImI and mutants.

				Generalized Born GB				Poisson-Boltzmann PB					
Mutant	ent^a^	vdw^a^	Coul^a^	epol^a^	SA^a^	ele^d^	des^e^	epol^a^	SA^a^	ele^d^	des^e^	ΔG^b^ (GB)	ΔG^c^ (PB)
S4A	29.2±1.4	−97.0±0.9	−266.7±9.1	294.8±7.5	−12.4±0.1	28.0±2.3	282.4±6.9	296.7±7.0	−12.1±0.1	30.0±2.5	284.6±6.6	−52.2±1.6	−50.0±2.1
D5A	26.6±1.6	−94.0±0.8	−435.0±8.0	463.7±7.4	−12.5±0.1	28.8±1.7	451.2±6.8	466.5±7.7	−12.3±0.1	31.5±1.7	454.7±7.1	−51.2±1.5	−48.2±1.6
D5N	25.4±1.2	−95.9±0.7	−432.1±9.2	462.7±8.6	−12.6±0.1	30.6±1.6	450.1±7.7	462.9±8.7	−12.2±0.1	30.8±1.7	450.8±7.7	−52.5±1.5	−51.9±1.6
D5K	26.0±2.2	−95.7±0.8	−634.4±9.9	674.2±9.5	−13.2±0.1	39.8±1.6	661.0±8.0	680.2±10.2	−12.6±0.1	45.8±2.4	667.6±8.8	−43.1±1.5	−36.5±±1.8
R7A	24.1±0.8	−86.3±0.6	−131.3±14.2	161.3±14.0	−11.2±0.1	30.0±2.5	150.1±15.5	166.2±13.4	−11.2±0.2	34.9±2.5	155.0±14.9	−43.3±1.6	−38.5±2.0
R7E	25.2±1.7	−89.8±0.8	−49.2±12.7	85.3±11.0	−12.3±0.1	36.2±2.9	73.0±12.5	104.2±11.2	−11.8±0.1	55.1±2.5	92.5±12.7	−40.7±2.2	−21.3±3.8
R7K	27.0±1.1	−93.3±0.8	−283.1±10.3	318.6±8.6	−12.4±0.1	35.5±2.4	306.1±9.2	322.4±8.5	−12.0±0.1	39.3±2.4	310.3±9.2	−43.2±1.4	−39.0±1.6
R7L	25.5±1.5	−90.9±0.7	−130.6±11.7	162.5±10.7	−11.8±0.1	31.9±2.3	150.7±11.6	170.1±10.8	−11.5±0.2	39.5±2.5	158.5±11.9	−45.4±1.7	−37.5±2.2
R7Q	26.7±1.3	−91.6±0.7	−136.3±10.7	166.7±9.2	−12.1±0.1	30.3±2.3	154.6±10.1	173.3±9.4	−11.8±0.1	37.0±2.4	161.6±10.3	−46.6±1.5	−39.6±1.8
A9S	25.5±1.3	−97.9±0.5	−280.9±8.9	305.7±7.7	−12.8±0.1	24.7±2.3	292.9±7.7	308.9±7.6	−12.2±0.1	28.0±2.6	296.7±7.89	−60.5±1.6	−56.7±2.1
W10A	25.2±1.2	−91.3±0.6	−261.4±9.0	289.6±8.0	−11.8±0.1	28.2±2.1	277.8±7.9	288.5±7.9	−11.4±0.1	27.0±2.3	277.1±7.9	−49.8±1.6	−50.6±1.8
W10F	26.1±1.7	−95.6±0.7	−260.9±8.9	290.6±7.8	−12.3±0.1	29.6±2.1	278.3±7.8	290.9±7.9	−11.9±0.1	30.0±2.2	279.0±8.1	−52.3±1.5	−51.5±1.6
W10T	27.1±1.7	−93.6±0.8	−267.2±8.8	296.2±7.7	−12.1±0.1	29.1±2.1	284.2±7.7	294.8±7.8	−11.7±0.1	27.6±2.3	283.1±7.7	−49.5±1.4	−50.6±1.7
W10Y	27.8±1.6	−96.1±0.9	−274.4±8.0	301.0±7.0	−12.7±0.1	26.6±2.2	288.4±6.9	302.3±7.0	−12.1±0.1	27.9±2.3	290.2±7.0	−54.3±1.6	−52.6±1.8
R11A	27.3±1.4	−95.0±0.8	−166.0±6.0	193.9±5.2	−12.1±0.1	27.8±1.7	181.8±5.3	197.3±4.9	−11.8±0.1	31.3±2.1	185.5±5.0	−52.0±1.3	−48.3±1.8
R11Q	27.1±1.6	−95.3±0.9	−168.6±6.3	197.9±5.6	−12.3±0.1	29.3±1.7	185.5±5.8	200.1±5.7	−12.0±0.1	31.5±2.0	188.1±5.9	−51.2±1.3	−48.6±1.6
IMI	24.7±1.2	−97.2±0.8	−271.2±8.2	299.4±7.1	−12.5±0.1	28.2±2.0	286.9±7.2	301.0±7.2	−12.1±0.1	29.8±2.1	288.9±7.3	−56.7±1.6	−54.7±1.8

a Decomposition of the binding free energy (ΔG) into entropy (ent), van der Waals (vdw), Coulombic electrostatic (Coul), polar desolvation (epol) and non-polar desolvation (SA) components. Two energy functions were used: molecular mechanics generalized Born (GB) surface area (MM-GB/SA) and molecular mechanics Poisson-Boltzmann (PB) surface area (MM-PB/SA). The temperature was set at 298K. The standard deviations of the binding free energies were computed using 15 frames.

b ΔG(GB) is the free energy computed using MM-GB/SA.

c ΔG(PB) is the free energy computed using MM-PB/SA.

d Electrostatic interaction (ele  =  epol + Coul).

e Desolvation contribution to the the binding free energy (des  =  epol + SA).

**Figure 9:**
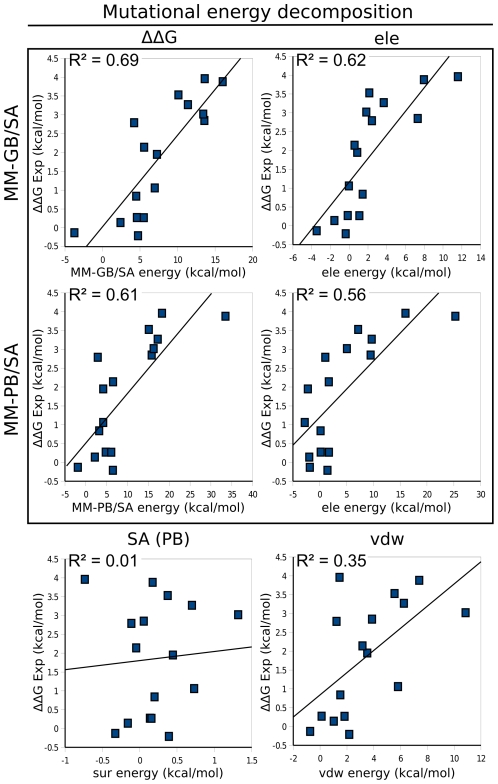
Correlation between calculated mutational energy components of ImI mutants and experimentally derived mutational energies. The explicit water minimization approach was employed to compute the Gibbs free energy (ΔΔG) using either molecular mechanics generalized Born (GB) surface area (MM-GB/SA) or molecular mechanics Poisson-Boltzmann (PB) surface area (MM-PB/SA) approaches at 298 K. The energies were decomposed into van der Waals (vdw), electrostatic (ele), surface area (SA, only shown for GB) and entropic components (not shown). Experimental mutational energies (ΔΔG Exp) were derived using the corresponding K_d_ values of ImI wild-type/α7-nAChR and ImI mutants/α7-nAChR [26]–[28].

The decomposition of the mutational free energies are displayed in Table 4 and the correlation between different contributions and experimentally derived mutational energies are shown in Figure 9. The electrostatic contribution has by far the best agreement with experimental energies (R^2^ = 0.62 with MM-GB/SA) and is therefore the major contributor to the specificity between mutants. The van der Waals interactions also participates, but to a lesser extent (R^2^ = 0.35). On the contrary, the hydrophobic effect does not allow differentiation of the mutants (R^2^ = 0.01). With a correlation coefficient of 0.23, the solute entropy term has only a small influence on specificity. Furthermore, the correlation coefficient between the experimentally derived and predicted mutational energies does not change substantially after including the solute entropic component (Figure 7 and 9). This justifies the proposal that solute entropic contributions, which are computationally demanding, can be neglected when predicting the ranking of single point mutant binding affinities based on the computation of binding energies.

**Table 4:** Decomposition of the mutational energies (kcal/mol) of ImI mutants.

			Generalized Born GB		Poisson-Boltzmann PB			
Mutant	vdw^a^	ent^a^	ele^a^	SA^a^	ele^a^	SA^a^	ΔΔG (GB)^b^	ΔΔG (PB)^c^
S4A	0.1±0.6	4.5±1.6	−0.2±0.2	0.1±0.1	0.2±0.2	0.0±0.2	4.6±1.4	4.8±1.5
D5A	3.2±0.6	1.9±1.4	0.6±0.3	−0.1±0.1	1.7±0.4	−0.2±0.1	5.6±1.8	6.5±2.1
D5K	1.5±0.8	1.3±1.0	11.6±1.6	−0.7±0.2	16.0±2.2	−0.5±0.2	13.6±1.6	18.3±3.3
D5N	1.2±0.6	0.7±1.8	2.4±1.0	−0.1±0.2	1.0±0.4	−0.1±0.1	4.2±1.3	2.9±1.8
R7A	10.9±0.7	−0.6±1.7	1.8±0.9	1.3±0.2	5.1±1.1	0.9±0.3	13.4±1.4	16.3±2.5
R7E	7.4±0.9	0.5±1.7	8.0±1.8	0.2±0.1	25.3±1.6	0.3±0.1	16.0±2.0	33.5±2.9
R7K	3.9±0.7	2.3±1.8	7.3±0.9	0.1±0.1	9.5±0.9	0.1±0.1	13.6±1.7	15.8±2.3
R7L	6.3±0.5	0.8±2.0	3.7±0.9	0.7±0.2	9.7±0.9	0.6±0.2	11.4±1.8	17.2±2.3
R7Q	5.6±0.7	2.0±1.7	2.1±1.1	0.4±0.1	7.2±1.0	0.4±0.1	10.1±2.3	15.1±1.8
A9S	−0.7±0.6	0.8±1.7	−3.5±1.1	−0.3±0.1	−1.8±0.4	−0.1±0.1	−3.7±1.9	−1.9±1.5
W10A	5.8±0.5	0.5±1.8	0.0±0.5	0.7±0.2	−2.8±0.3	0.7±0.2	7.0±1.7	4.2±1.1
W10F	1.5±0.5	1.4±2.2	1.4±0.3	0.2±0.1	0.2±0.3	0.2±0.1	4.5±1.3	3.2±1.4
W10T	3.5±0.6	2.4±2.3	0.9±0.4	0.4±0.1	−2.2±0.5	0.4±0.1	7.3±2.4	4.2±2.4
W10Y	1.0±0.6	3.1±1.9	−1.6±0.9	−0.2±0.1	−2.0±0.4	0.0±0.1	2.4±2.0	2.2±1.9
R11A	2.2±0.5	2.6±1.2	−0.4±0.9	0.4±0.2	1.4±1.0	0.3±0.2	4.7±1.3	6.5±1.7
R11Q	1.8±0.6	2.4±1.8	1.1±0.8	0.2±0.1	1.7±0.8	0.1±0.1	5.5±1.9	6.1±1.9

a Decomposition of the mutational energy (ΔΔG) into van der Waals (vdw), entropic (ent), electrostatic (ele) and non-polar desolvation (SA) components. Two energy functions were used: molecular mechanics generalized Born (GB) surface area (MM-GB/SA) and molecular mechanics Poisson-Boltzmann (PB) surface area (MM-PB/SA). The temperature was set at 298K. The standard deviations of the mutational energies and energy components were computed using 15 frames.

b ΔΔG(GB) is the mutational free energy computed using MM-GB/SA.

c ΔΔG(PB) is the mutational free energy computed using MM-PB/SA.

### Concluding remarks

In this study, an extensive computational analysis of a complex between an α-conotoxin, ImI, and an nAChR, α7-nAChR, was carried out. In the absence of the crystal structure of a complete nAChR extracellular domain, modeling the interaction of an inhibitor of an nAChR is difficult. Nevertheless, we successfully studied the binding mode between α-conotoxin ImI and α7-nAChR using a combination of comparative modeling and molecular dynamics simulation. Using this model, we have explained the effect of mutations described in previous experimental studies.

The structures of 16 mutated ImI/α7-nAChR complexes were refined using MBA or MDBA, and the binding energies were predicted using MM-PB/SA and MM-GB/SA. To our knowledge, this study constitutes the first attempt to use these energy functions to study the binding of a range of α-conotoxin variants to an nAChR. The approach using a simple minimization to refine the model (MBA) led to the best agreement between predicted mutational energies and experimental values.

Another important conclusion of our study is that affinity between ImI analogues and α7-nAChR was mainly governed by van der Waals and non-polar desolvation energies, whereas the electrostatic interactions were mainly important for the specificity. Interestingly, the entropy had little influence on the mutational energy of single point mutants. Because α-conotoxins share the same tightly packed structural fold, our observations on the energy decomposition are likely to help in the rational optimization of α-conotoxins pharmacological properties in general.

In order to perform extensive computational scanning of α-conotoxins, a fast and accurate approach is necessary. In this respect, we have identified that the best method to achieve this goal is to refine the mutated models by minimization using explicit solvation and to compute mutational energies using MM-GB/SA.

## Materials and Methods

### Comparative modeling of α7-nAChR

In the absence of the crystal structure of α7-nAChR, the human α7-nAChR LBD was modeled using a comparative approach, following a strategy described previously [51]–[53]. The crystal structure of an isolated *Mus musculus* muscle type extracellular domain of the α1-nAChR subunit in complex with the inhibitor α-bungarotoxin was solved at 1.94 Å resolution (PDB ID: 2qc1) [35]. The *Mus musculus* α1-nAChR subunit shares 38% sequence identity with the *Homo sapiens* α7 subunit and superimposes with, on average, 2.9 Å rmsd with the AChBP subunits. An electron microscopy structure of a complete muscle type nAChR of *Torpedo marmota* (PDB ID: 2bg9) revealed a similar arrangement of subunits as the one presented by AChBP. As the electron microscopy structure is of low resolution (4 Å), the AChBP structures (PDB ID: 2c9t) were employed as structural templates in our comparative modeling strategy to orient the five α7 subunits in the pentamer. The orientations of the side chains were modeled according to the α1 template (PDB ID: 2qc1) due to its overall higher sequence identity to the α7 subunit than AChBP. A sequence alignment between the two structural templates and α7-nAChR LBD is displayed in Figure 2. The secondary structure elements and ligand-binding sites observed on the experimental structures and predicted for α7-nAChR are also shown in Figure 2. The modeling of the nAChR C-loop required special attention as its change in conformation allows the binding site to accommodate ligands of different sizes [33]. In our model, the structure of AChBP in complex with ImI (PDB ID: 2c9t) was used to derive restraints in the C-loop region because AChBP has locally higher sequence identity than α1, and because the C-loop conformation in the AChBP structure allows ImI to fit in the binding site. Conversely, the Cys-loop, the β1-β2 loop, the A-loop, and the B-loop were modeled using information from the α1 template because it displays higher sequence identity than AChBP. Multiple sequence alignment between *Aplysia californica* AChBP and the LBD of α1, α2, α3, α4, β1, β2, α6, α7, α9 and α10 was generated using MUSCLE with default parameters [54]. The multiple alignment between AChBP, α7 and α1 was then manually adjusted based on structural superimpositions of the crystal structures of AChBP (PDB ID: 2c9t) and α1 (PDB ID: 2qc1). The comparative modeling program Modeller [55] (version 9v7) was then employed to generate 100 three-dimensional structural models of the α7-nAChR complex. The Cys-loop region was modeled using the α1 subunit template only, whereas the C-loop and B-loop were modeled based on AChBP. The model selected according to the DOPE score [56] was analyzed using MolProbity [57] and 94% residues were in the favorable region of the Ramachandran plot, which is acceptable for a comparative model [56].

### ImI/α7-nAChR complex

A model of the structure of the complex ImI/α7-nAChR was obtained by comparative modeling. An X-ray diffraction structure of the complex between ImI and AChBP (PDB ID: 2c9t) was used to provide restraints between ImI and the nAChR and also structural restraints to ImI conformation. The structure of α7-nAChR was modeled using the same sequence alignment/structure described previously to model the apo state. The use of a comparative modeling approach is justified by the fact AChBP and α7-nAChR are likely to have very similar binding modes because they share a high level of sequence identity in their binding sites (52% identity according to alignment in Figure 2).

### Molecular dynamics simulation

Molecular dynamics simulations (MD) were performed using Gromacs 3.3.1 package [55] and the 53a6 forcefield. The ImI/α7-nAChR model was solvated in a cubic box with an edge length of 11.4 nm solved by adding 40,773 SPC water molecules. 74 Na^+^ and 27 Cl^−^ ions were added to simulate a physiological NaCl concentration of 0.1 M and to neutralize the system. The system was minimized using 1000 steps of steepest descent algorithm. The temperature was progressively raised from 0 K to 300 K over 100 ps of constant pressure and temperature (NPT) MD simulation with all the protein atoms restrained to their initial position. Ten nanosecond NPT MD was then performed on the whole system without restraints with Berendsen temperature bath coupling set at 300 K and an isotropic molecule based scaling setup at 1 atm [58]. The electrostatic interaction between non-covalent atoms was computed with particle-mesh Ewald method [59] with a distance cutoff of 10 Å. The LINCS algorithm [60] was used to constrain all bonds and the time step of the simulation was set to 2 fs. The simulation of the apo state α7-nAChR was prepared using the same procedure. Ten nanosecond MD simulations were performed twice for ImI/α7-nAChR and three times for the α7-nAChR in apo state systems. Stability and conformational variabilities of those five simulations are provided in Figure 3 and in supplementary material figure S1.

### Models of single point mutants

In the MBA, 15 frames were extracted every 500 ps in the interval between 3–10 ns of the 10 ns molecular dynamics simulation trajectory of the wild-type model. Each frame was minimized using AMBER10 [61] (with the AMBER ff03 forcefield) by 2000 steps of steepest descent algorithm followed by 2000 steps of conjugate gradient algorithm with the backbone of the complex restrained. Two thousand five hundred steps of steepest descent minimization and 2500 steps of conjugate gradient minimization were then performed without restraints. The side chains in the ligand were mutated using Modeller and all the residues (including residues of the ligand) were minimized using AMBER10 [61]. In the DDDCM approach, ε = 4r was used, whereas in the EWM approach, the protein was solvated in a water box with a minimum of 8 Å between the solute and the side of the box.

In the MDBA, a water cap with a radius of 16 Å from the center of the binding pocket was added. MD was only performed on the mutants of the last frame obtained from MBA above. Before performing MD, the system was minimized using 2000 steps of steepest descent minimization followed by 2000 steps of conjugate gradient minimization. The water box was equilibrated by increasing the temperature from 0 to 300 K while maintaining the solute under constraints, and then further maintaining the simulation at 300 K for 40 ps. In the production phase, the restraints in the binding site were removed and 500 ps MD was performed with a 2 fs time step. The non-bonded cutoff was set to 12 Å and SHAKE was applied for all the bonds involving hydrogen atoms. For strategy (i), water molecules and residues within 6 Å of the ligand were flexible; for strategy (ii), water molecules and residues within 4.5 Å of the ligand were flexible; for strategy (iii), water molecules and residues within 6 Å of the mutated residues were flexible; for strategy (iv), all the atoms of the ligand and water molecules were flexible; and for strategy (v), only the water molecules were flexible. For every strategy, 50 frames were extracted every 2 ps in the interval comprised between 400–500 ps of the 500 ps MD simulation.

### Additional MD simulations of some mutants

To provide qualitative explanations to the effect of the mutations of ImI-D5N, ImI-R7Q, α7-Q117A, α7-R186V, α7-N111S, α7-S113A, α7-P120A and α7-G153S, additional MD were performed for at least 500 ps. In those MD, a similar water cap was used, as described previously, and residues within 6 Å of the mutated side chain were flexible.

### Computation of mutational energy

The values of the binding free energy (ΔG _binding_) for each mutant were calculated based on the following equation:
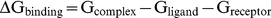
(1)


The free energy can be decomposed into three components:
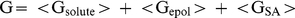
(2)where G _solute_ is the solute Gibbs free energy, G _epol_ represents the polar contribution to the solvation energy and G _SA_ represents non-polar contribution to the solvation energy. Polar contribution to the solvation energy is determined by solving the Poisson-Boltzmann Equation using the PB module implemented in AMBER10 [61], or the GB approach implemented in AMBER10 [62]. The non-polar contribution to the solvation energy is calculated using:
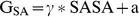
(3)where solvent-accessible surface area (SASA) was determined using the Molsurf [63] algorithm with a probe radius of 1.4 Å. The surface tension γ and constant parameter a in equation (3) were taken to their default values 0.0072 kcal/mol^−1^ Å^2^ and, 0 kcal/mol^−1^ Å^2^ respectively. The effect of residue mutation on the binding energy was computed using:
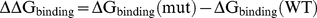
(4)where ΔΔG _binding_ was defined as mutational energy that is the binding energy difference between the wild-type ligand (ΔG _binding_ (WT)) and its mutants (ΔG _binding_ (mut)).

The entropy contribution was estimated using normal-mode analysis, which employed the atomic fluctuation matrix produced from a normal mode calculation [64]. Alternatively, for some calculations, we made the approximation that the wild-type and mutated complexes have similar entropies. Using this approximation:
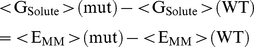
(5)where E _MM_ is the molecular mechanical energies of the proteins as given by the molecular mechanics potential. This equation was used to compute the difference of internal Gibbs free energy for the complex, the ligand and the receptor. The binding free energy of the mutants was calculated by solving equations (4) and (5) using the MMPBSA.py script, which is part of the AMBER10 distribution. The Poisson-Boltzmann equations were solved using internal dielectric and external dielectric constants set to 2.0 and 80.0, respectively, a probe radius of 1.4 Å, a grid spacing set to 0.5 Å and ionic strength set to 0.15 M/L. For the GB algorithm, the salt concentration was set to 0.15 M/L.

## Supporting Information

Figure S1 Rmsd, Rmsf and distance plots of the apo-state model and ImI/α7-nAChR complex over the 10 ns molecular dynamics simulations. Three α7-nAChR apo-state and two α7-nAChR/ImI simulations were performed in total. In the first row, β strand α carbon root-mean-square deviations (RMSD) of each of the subunits over the molecular dynamics simulations to the starting frame. In the second row, α carbon root-mean-square fluctuation (RMSF) of each subunit over the 10 ns molecular dynamics simulation ensemble. In the third row, fluctuation of the distance between the sulfur atom of α7-C190 side chain and the α carbon of α7-Y32. This distance characterizes the closure of the C-loop.
(TIFF)

Figure S2 Correlation between the experimentally derived mutational energies and calculated mutational energies of ImI and receptor mutants. Mutational energies were computed using either molecular mechanics generalized Born (GB) surface area (MM-GB/SA) or molecular mechanics Poisson-Boltzmann (PB) surface area (MM-PB/SA) energy functions at 298 K. The mutated models were refined using MBA with either distance dependent dielectric constant minimization (DDDCM) or explicit water minimization (EWM).
(TIFF)
