# Supplementary figures and images for: Blockade of Neuronal α7-nAChR by α-Conotoxin ImI Explained by Computational Scanning and Energy Calculations
Source: PLoS Comput Biol. 2011 Mar 3;7(3):e1002011. doi: 10.1371/journal.pcbi.1002011 (PMC3048385; doi:10.1371/journal.pcbi.1002011)

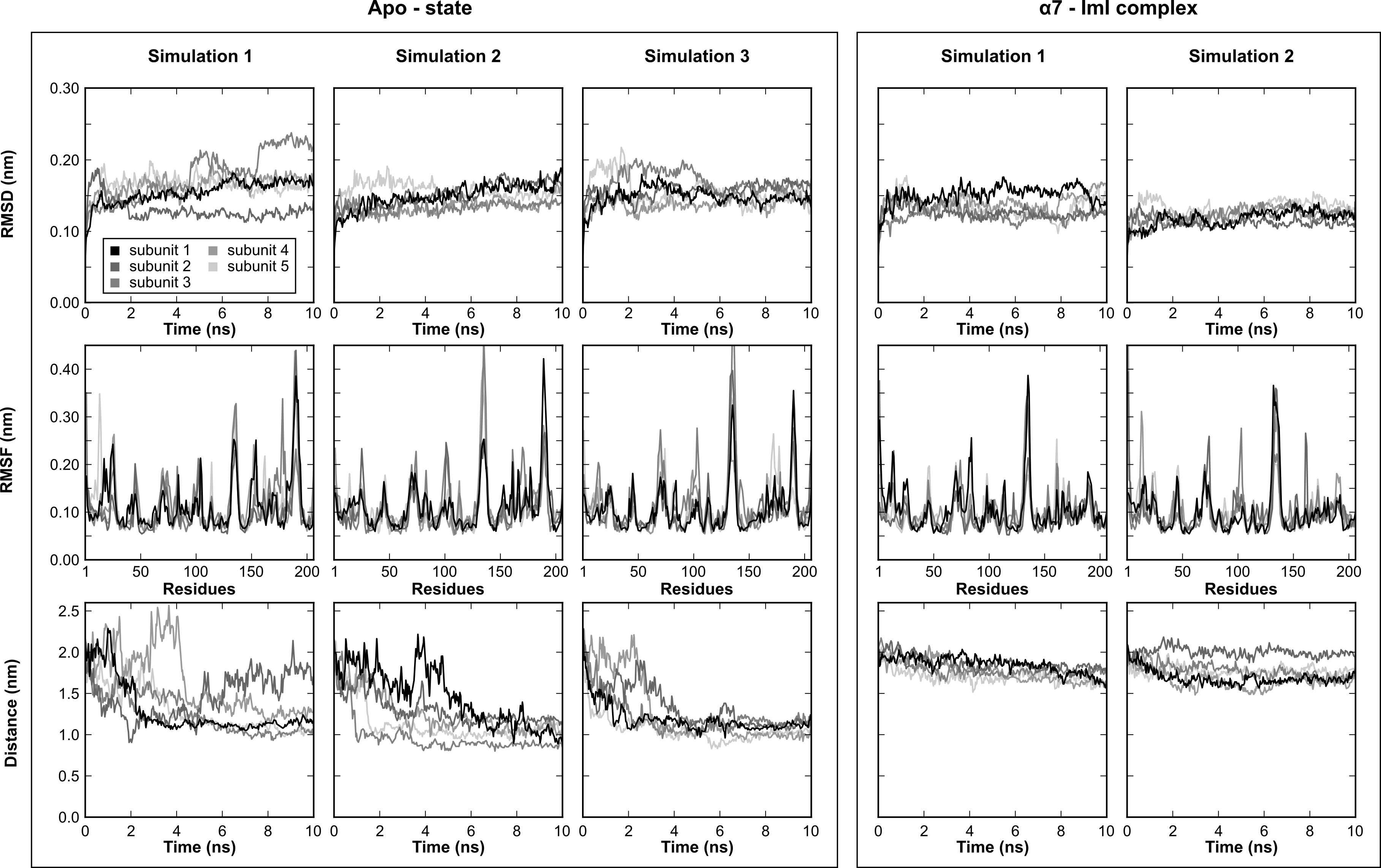

Supplement: Figure S1 — Rmsd, Rmsf and distance plots of the apo-state model and ImI/α7-nAChR complex over the 10 ns molecular dynamics simulations. Three α7-nAChR apo-state and two α7-nAChR/ImI simulations were performed in total. In the first row, β strand α carbon root-mean-square deviations (RMSD) of each of the subunits over the molecular dynamics simulations to the starting frame. In the second row, α carbon root-mean-square fluctuation (RMSF) of each subunit over the 10 ns molecular dynamics simulation ensemble. In the third row, fluctuation of the distance between the sulfur atom of α7-C190 side chain and the α carbon of α7-Y32. This distance characterizes the closure of the C-loop. (TIFF) [file pcbi.1002011.s001.tiff]

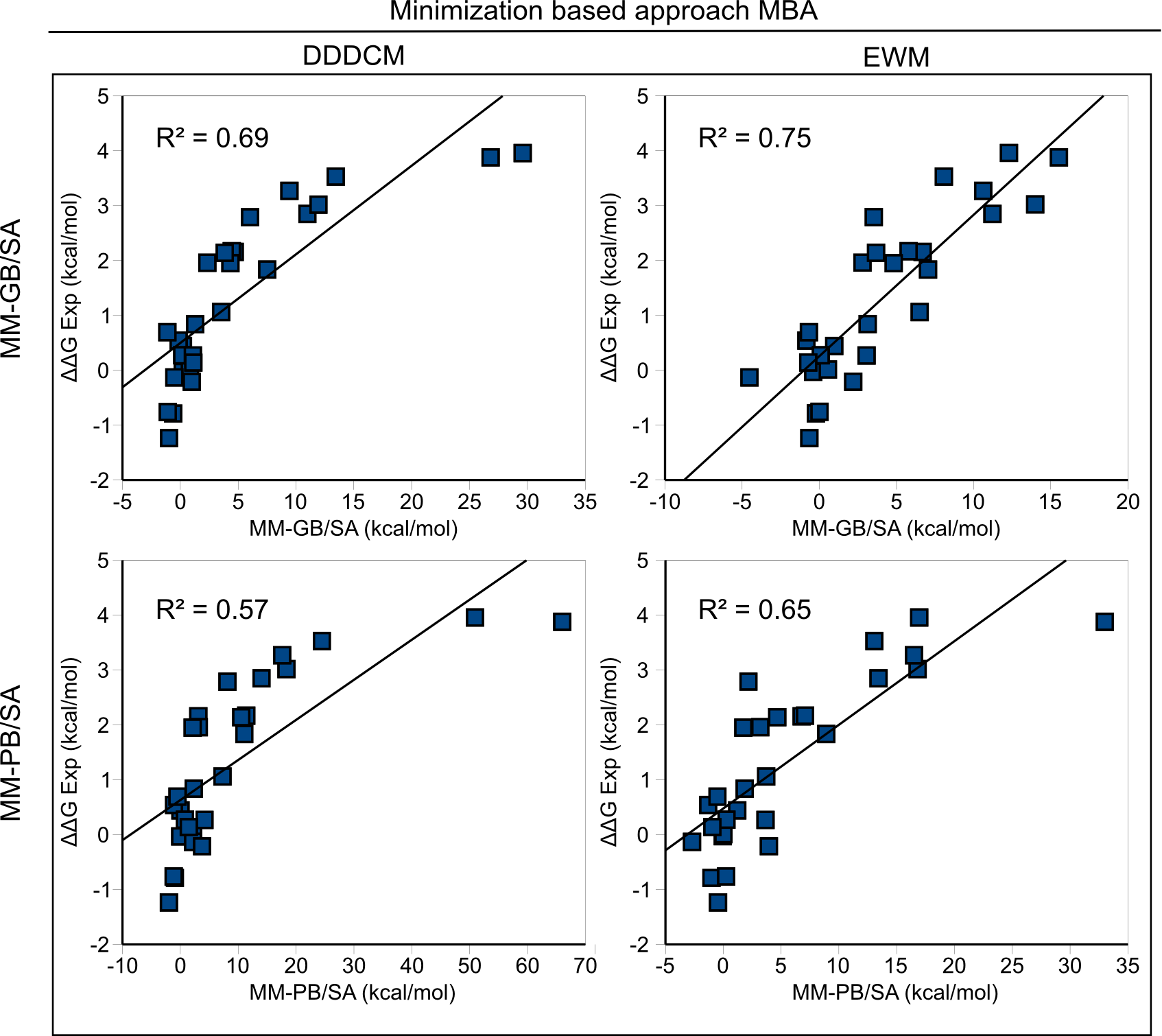

Supplement: Figure S2 — Correlation between the experimentally derived mutational energies and calculated mutational energies of ImI and receptor mutants. Mutational energies were computed using either molecular mechanics generalized Born (GB) surface area (MM-GB/SA) or molecular mechanics Poisson-Boltzmann (PB) surface area (MM-PB/SA) energy functions at 298 K. The mutated models were refined using MBA with either distance dependent dielectric constant minimization (DDDCM) or explicit water minimization (EWM). (TIFF) [file pcbi.1002011.s002.tiff]
